# Associations of CYP2B6, CYP2C19, and CYP2D6 isoforms and phenoconversion with sertraline pharmacokinetics

**DOI:** 10.1371/journal.pone.0351241

**Published:** 2026-06-10

**Authors:** Begoña Tapia-Alzuguren, Covadonga Canga-Espina, Patricio Molero, Azucena Aldaz

**Affiliations:** 1 Pharmacy Services, Clínica Universidad de Navarra, Pamplona, Navarre, Spain; 2 Department of Psychiatry and Clinical Psychology, Clínica Universidad de Navarra, Pamplona, Navarre, Spain; 3 Instituto de Investigación Sanitaria de Navarra (IdiSNA), Pamplona, Navarre, Spain; University of Iowa, UNITED STATES OF AMERICA

## Abstract

Interindividual variability in sertraline exposure is substantial and may be influenced by genetic variation in drug-metabolizing enzymes as well as by concomitant medications that modify enzyme activity. This study explored the association between genetic variability in the cytochrome P450 isoforms CYP2B6, CYP2C19, and CYP2D6 and sertraline pharmacokinetics in a real-world clinical setting, accounting for medication-related phenotype modification. We conducted an observational case-series analysis including seven hospitalized patients with available pharmacogenetic data and sixteen therapeutic drug monitoring measurements of sertraline. Pharmacokinetic parameters were descriptively compared across genotype-predicted metabolic phenotypes and after adjustment for concomitant medications with potential inhibitory or inducing effects, as informed by available interaction data. Before accounting for medication-related phenotype modification within a descriptive framework, variability in sertraline exposure appeared to follow patterns consistent with genotype-predicted phenotypes related to CYP2B6, whereas limited or inconsistent patterns were observed for CYP2C19 and CYP2D6. After incorporating co-medication effects, patterns across sertraline serum concentrations, concentration-to-dose ratios, and apparent clearance were more consistent for adjusted CYP2B6 phenotypes. Adjusted CYP2C19 phenotypes showed additional variability patterns in dose-normalized exposure and clearance, while no consistent associations were observed for CYP2D6 after adjustment. The metabolite-to-parent compound ratio showed no clear relationship with either genetic or adjusted phenotypes. These findings suggest that interpretation of pharmacogenetic information for sertraline may benefit from integrating CYP2B6 and CYP2C19 genotype data with concomitant medication use. In this exploratory case series, accounting for medication-related phenotype modification revealed variability patterns that were less evident when genetic information was considered alone. Current guidelines consider CYP2B6 and CYP2C19 actionable for sertraline; our findings suggest that integrating co-medication–informed interpretation with pharmacogenetic testing and therapeutic drug monitoring may further refine individualized assessment of sertraline exposure in complex clinical settings.

## Introduction

Approximately two-thirds of patients fail to achieve full remission following initial antidepressant treatment, increasing the risk of recurrence and treatment resistance [1]. This substantial interindividual variability in treatment outcomes has stimulated growing interest in precision medicine approaches aimed at individualizing pharmacotherapy [2]. In this context, pharmacogenetic testing provides information on genetically determined enzyme activity, whereas pharmacokinetic monitoring characterizes the resulting drug exposure under real-world conditions, integrating both genetic and non-genetic influences [3].

Although genotyping offers stable, environment-independent data, its clinical interpretation is challenged by numerous factors that contribute to variability in drug concentrations, as consistently reported for sertraline [4–10]. Co-medication, diet, smoking status, and other patient-specific factors can modify enzyme activity and lead to discrepancies between genotype-predicted metabolism and observed drug exposure, a phenomenon commonly referred to as phenoconversion [11–16]. Phenoconversion should be understood as a descriptive clinical concept reflecting altered metabolic capacity in a given context, rather than as a uniform or directly measurable biological state.

In clinical pharmacology, pharmacokinetic monitoring is widely used to characterize drug exposure for compounds with high interindividual variability. At identical doses, serum concentrations may differ substantially due to a combination of genetic variability, age, sex, body weight, adherence, absorption, and drug–drug interactions [3]. In psychiatry, pharmacokinetic monitoring —as one of the most established and clinically implementable tools for treatment individualization— has also been shown to support dose optimization, aid in the identification of non-adherence or malabsorption, and may improve the cost-effectiveness of treatment strategies [3,17].

Selective serotonin reuptake inhibitors (SSRIs) are recommended as first-line pharmacological treatment for moderate to severe major depressive disorder because of their favorable efficacy–safety balance [18–22]. Sertraline is among the most frequently prescribed SSRIs, owing to its broad clinical use, limited sedative effects, and overall tolerability [18]. Despite this favorable profile, marked interindividual variability in sertraline serum concentrations has been repeatedly documented [23–27].

Sertraline undergoes extensive hepatic metabolism involving multiple cytochrome P450 isoforms. *In vitro* data support the contribution of several enzymes to its biotransformation, including CYP2B6, CYP2C19, CYP3A4, CYP2C9, and CYP2D6, reflecting a distributed metabolic pathway, including the formation of N-desmethylsertraline (N-SER), a metabolite not considered clinically active. Among these, CYP2B6 and CYP2C19 appear to play a more prominent role in explaining interindividual variability *in vivo*, whereas the contribution of other isoforms such as CYP3A4 may be more context-dependent [23–25,28]. Although sertraline is administered as the single cis-(1S,4S) enantiomer, stereoselective substrate recognition across multiple CYP enzymes may contribute to distributed metabolism and attenuate the magnitude of single-enzyme effects *in vivo*. CYP2B6 exhibits substantial functional variability and contributes to the metabolism of a meaningful proportion of commonly used drugs [26]. Reduced-function alleles (CYP2B6*6, CYP2B6*18) are more common in African and South Asian populations, while increased-function CYP2B6*4 is prevalent in South Asians. Homozygous reduced-function alleles may be associated with markedly decreased enzyme activity, whereas heterozygous carriers often show intermediate metabolic capacity. In Europeans, 61% carry *wild-type* alleles with normal activity [27,29].

CYP2C19 is likewise highly polymorphic. In Europeans, approximately 59% carry two CYP2C19*1 (normal function), 18% carry the non-functional *2 allele, and 22% the increased-function *17 allele, leading to rapid (*1/*17) or ultrarapid (*17/*17) metabolism [29]. By contrast, available evidence suggests that CYP2D6 polymorphisms have limited influence on sertraline exposure [25,30], despite substantial global phenotypic diversity, including reduced-function (*17, *29) alleles frequent in African populations, the non-functional *4 allele common in Europeans, and increased-function (1xN) more prevalent in Oceanic populations [31]. No relevant evidence links CYP3A4 variants to sertraline pharmacokinetics [28].

Evidence on the impact of these variants is mixed: CYP2B6 polymorphisms may moderately affect sertraline metabolism, and some studies find CYP2C19 variants irrelevant [32], while others report that reduced-function CYP2C19 alleles significantly increase concentrations [33,34]. Increased-function alleles appear less clinically significant [35]. Notably, much of the existing evidence derives from heterogeneous study designs, and only limited data explicitly address the modifying role of concomitant medications in naturalistic clinical settings.

Given these uncertainties, the present study was designed as an exploratory case series to descriptively assess the relationship between CYP2B6, CYP2C19, and CYP2D6 genotypes, concomitant medication use, and observed sertraline pharmacokinetic parameters in a naturalistic clinical context. By focusing on individual patient trajectories and real-world therapeutic drug monitoring data, this study aims to generate hypothesis-generating insights into the interplay between pharmacogenetics, phenoconversion, and sertraline exposure, without attempting causal inference or population-level estimation.

## Materials and Methods

### Setting and study population

This retrospective sub-cohort analysis forms part of the PREDEP-SERT project, a single-centre, observational, longitudinal, ambispective study investigating the relationship between sertraline blood concentrations and its effectiveness and safety in major depressive disorder (REec: 0014–2022-OBS). Study design, methods, and participant characteristics have been described elsewhere [36]. The present analysis is explicitly conceived as an exploratory, descriptive case series, focusing on pharmacokinetic observations in a real-world clinical setting rather than on population-level inference or causal assessment.

Data were obtained from pharmacokinetic records of patients treated in the Department of Psychiatry, Clínica Universidad de Navarra, generated upon psychiatrist request for sertraline monitoring. Eligible patients had depressive symptoms (with or without psychotic features) from diverse psychiatric or medical diagnoses (excluding isolated dysthymia), were treated with sertraline, and underwent concurrent pharmacokinetic and pharmacogenetic testing between August 2018 and January 2022. In our clinical setting, pharmacogenetic testing is not performed routinely but is requested on a case-by-case basis, typically in patients with suspected treatment resistance, adverse effects, or potential drug–drug interactions. Additionally, as these tests are not systematically covered and are often patient-funded, their use remains limited. Consequently, only a small number of patients fulfilled all inclusion criteria during the study period.

Data were accessed for research purposes between 13/11/2022 and 25/05/2023. These evaluations informed dosage adjustments based on clinical judgment, particularly in the presence of drug interactions. Steady state was operationally defined as a clinically stable dosing period of at least five days prior to sampling, consistent with the expected pharmacokinetic profile of sertraline (half-life approximately 24–32 hours) and real-world therapeutic drug monitoring practice; suspected non-adherent patients were excluded. Because multiple pharmacokinetic determinations could be available for a given patient, repeated observations were intentionally retained to reflect real-world clinical trajectories and intra- and inter-individual variability, rather than being aggregated into a single summary value.

The confidentiality of the subjects’ personal data was protected in accordance with applicable regulations. The project was approved by the local Research Ethics Committee and was conducted in accordance with the Declaration of Helsinki (EO_2021/19). This study used pseudonymized clinical data and was exempted from informed consent under Spanish Regulation RD 957/2020.

### Therapeutic drug monitoring

The monitoring protocol, developed by the Clinical Pharmacokinetics Unit (CPU) of the Pharmacy Service, followed current evidence and AGNP-TDM guidelines [3].

Steady-state serum samples were obtained from 3–5 mL of venous blood collected into gel-free Vacutainer tubes. Sampling was performed at trough (Cmin) under steady-state conditions (≥5 elimination half-lives), approximately 24 hours post-dose (acceptable window: 24 ± 4 hours). Samples obtained before 20 hours post-dose were excluded from the analysis. Following centrifugation, serum was processed the same morning. If immediate processing was not feasible, samples were stored at 2–8°C, protected from light, and analyzed within 24–72 hours. The quantification of serum concentrations of sertraline and its metabolite N-SER was performed via ultra-high performance liquid chromatography with tandem mass spectrometry (UPLC-MS/MS) at the CPU, employing the ChromeSystem commercial analysis kit (MassTox® TDM Series A Parameter Set Antidepressants 1/Extended). This commercial kit comprised extraction and precipitation reagents, an internal standard, calibrators and quality controls, mobile phases, and a chromatographic column. The chromatographic equipment employed was a Waters Acquity™ quadrupole which utilized a positive electrospray ionization technique for the mass spectrometry.

Calibration preceded each run. Detection transitions were m/z 306.2 → 159 (sertraline) and 201 → 129.1 (N-SER). Recovery rates were 93–101% for sertraline and 96–106% for N-SER. Limits of quantification were 0.75 ng/mL for sertraline (linear to 450 ng/mL) and 6.00 ng/mL for N-SER (linear to 750 ng/mL). Intra-assay coefficients of variation ranged from 3.6% to 8.6% and from 4.8% to 8.5%, respectively, while inter-assay coefficients ranged from 5.0% to 6.3% and from 6.6% to 6.9%.

### Genotyping

Pharmacogenetic analysis was performed by OneOme LLC (Minneapolis, MN, USA) for CYP2B6, CYP2C19, and CYP2D6 alleles (Table 1) via real-time polymerase chain reaction (PCR) using methods based on Thermo Fisher TaqMan® and/or LGC Biosearch BHQ® probes. CYP2D6 copy number analysis targeted promoter, intron 2, intron 6, and exon 9 sequences, detecting deletions, duplications, multiplications, and hybrid alleles, but not distinguishing duplications in the presence of deletions.

**Table 1:** CYP450 enzyme isoforms and polymorphisms analyzed.

CYP450 enzyme isoforms	Polymorphisms analyzed
CYP2B6	*4, *5, *6, *7, *9, *16, *18
CYP2C19	*2, *3, *4, *4B, *10, *17
CYP2D6	*2, *2A, *3, *4, *4M, *4N, *5, *6, *6C, *7, *8, *9, *10, *11, *12, *13, *14, *15, *17, *18, *19, *20, *29, *31, *34, *35, *36, *39, 41, *42, *59, *63, *64, *65, *68, *69, *70, *91, *109, *114

### Genotype-predicted phenotype

Theoretical metabolic phenotypes were assigned according to allele activity following published recommendations, with allele nomenclature based on PharmVar and phenotype classification consistent with CPIC guidelines, including consideration of structural variants (e.g., gene duplications and hybrid alleles) where applicable [23,37]. These categories represent predicted enzymatic capacity inferred from genotype and do not constitute direct measurements of *in vivo* metabolic activity. Patients were classified as poor metabolizers (PM_t_) when two non-functional alleles were present, as in CYP2B6*6/*6, CYP2C19*2/*2, or CYP2D6*4/*4 + *68. Intermediate metabolizers (IM_t_) carried one functional or increased-function allele together with a deficient or non-functional allele, such as CYP2B6*1/*6, CYP2C19*1/*2, or CYP2D6*1/*4. Normal metabolizer (NM_t_) had two functional alleles, including CYP2B6*1/*1 (*wild-type*) and *1/*5, CYP2C19*1/*1 (*wild-type*), or CYP2D6*1/*2A, *1/*35, *1/*10, and *1/*10 + 36. Rapid metabolizer (RM_t_) carried one functional and one increased-function allele, as in CYP2C19*1/*17, while ultrarapid metabolizers (UM_t_) had two increased-function alleles or increased gene copy number, such as CYP2C19*17/*17 or CYP2D6*1/*2Ax2. No increased-function CYP2B6 alleles were identified in this cohort.

### Phenoconversion

Concomitant medications were reviewed to identify potential modifiers of enzymatic activity. Perpetrator classification was based on established interaction resources (FDA and Flockhart tables) [12,38], complemented by literature evidence where necessary, and categorized according to level of supporting evidence. Phenoconversion was considered as a descriptive clinical framework reflecting the potential modification of genotype-predicted metabolism by co-medications, based on published guidance [11,13]. Moderate inhibitors were considered capable of reducing predicted metabolic activity by one category, whereas strong inhibitors were considered capable of reducing activity to a poor metabolizer phenotype (PM_p_). Inducers were considered capable of increasing predicted activity by one category, and potent inducers were considered capable of converting normal metabolizers (NM_t_) into ultrarapid metabolizers (UM_p_). Extreme phenotypes, such as PM_t_ in the presence of inhibitors or UM_t_ in the presence of inducers, were not adjusted due to absence of lower or higher phenotype categories, respectively. In cases where both inhibitors and inducers were present, no adjustment was applied due to insufficient evidence. These phenotype reclassifications were applied conceptually to support clinical interpretation and hypothesis generation and were not intended to represent quantitative or definitive measurements of enzymatic activity. Serum concentration data were therefore interpreted as observed pharmacokinetic outcomes rather than as direct proxies of metabolic phenotype.

### Data collection

Collected variables included demographic and anthropometric data (sex, age, height, weight, body mass index (BMI)); clinical variables (length of hospital stay, smoking status); pharmacological variables (sertraline dose, duration of treatment at stable dose, concomitant medications, including substance, dose, and frequency); and pharmacokinetic variables (sampling date and time, serum concentrations of sertraline (CSER) and its metabolite N-SER, concentration-dose ratio (CDR), weight-normalized CDR (CDR/kg), apparent total clearance (CL/F) based on steady-state assumptions, and metabolite-to-parent ratio (N-SER/SER)). Sertraline concentrations were measured as total (bound + unbound) serum concentrations; unbound concentrations were not available. Accordingly, pharmacokinetic parameters, including apparent clearance (CL/F), reflect total drug exposure. All patients received sertraline in tablet formulation; no oral solution was used and feeding status was not systematically standardized or recorded, reflecting routine clinical practice in a naturalistic inpatient setting. Psychiatric diagnoses and comorbidities were documented according to ICD-10 and DSM-5 criteria current at the time of data collection.

### Data analysis

All analyses were performed in Stata® version 15.1. Variable normality was assessed using the Shapiro–Wilk test, visual inspection, skewness, and kurtosis. Normally distributed variables were expressed as mean ± standard deviation (SD), while non-normally distributed variables were summarized as median and interquartile range (IQR). Given the exploratory case-series design, small sample size, and inclusion of repeated observations within individuals, analyses were limited to non-parametric, descriptive group comparisons. Mann–Whitney U tests were used for comparisons between two groups, and Kruskal–Wallis tests for comparisons involving more than two groups. P-values are reported solely as descriptive measures to highlight patterns in the data and should be interpreted cautiously. They do not imply independence of observations, statistical inference at the population level, or causal associations. A two-sided p-value <0.05 was considered indicative of potentially relevant differences warranting further investigation in future studies. Sample sizes reported in tables generally reflect the number of sertraline measurements rather than the number of individual patients, except in Table 3, where counts refer to patients. Sertraline concentrations were interpreted in relation to the AGNP consensus therapeutic drug monitoring guidelines for antidepressants, which propose a reference range of 10–150 ng/mL for descriptive clinical interpretation [3].

## Results

### Study population

This study analyzed 16 sertraline pharmacokinetic measurements obtained from seven individual patients who met pharmacogenetic testing criteria. Patient characteristics, including CYP2B6, CYP2C19, and CYP2D6 genotypes and predicted phenotypes, demographic and anthropometric variables, smoking status, pharmacokinetic parameters, psychiatric diagnoses, and concomitant medications, are summarized in Table 2. Measurements were obtained in a hospital setting with equal distribution by sex (8 measurements in females and 8 in males). Mean length of hospitalization was 48.7 ± 17.4 days; mean age was 50.3 ± 21.5 years (47.5 ± 19.4 in females; 53 ± 24.4 in males). Mean height was 168 ± 9.1 cm (161 ± 8.7 cm in females; 175 ± 1.9 cm in males), mean body weight was 69.5 ± 13.2 kg (62 ± 12.4 kg in females, 77 ± 9.4 kg in males), and mean BMI was 24.8 ± 5.3 kg/m² (24.27 ± 7.2 kg/m² in females; 25.23 ± 2.8 kg/m² in males). Most patients were non-smokers (75%). Among those reporting active smoking (25%), only one patient reported daily cigarette consumption (7 cigarettes/day).

**Table 2:** Characteristic of the study population [n = 16 sertraline measurements].

Patient under analysis (n = 7)		A	B	C	D	E	F	G
**Number of pharmacokinetic measurements (n = 16)**		n = 2	n = 1	n = 2	n = 1	n = 3	n = 5	n = 2
**Genotype (Theoretical enzymatic phenotype)**	**CYP2B6**	*6/*6 (PM_t_)	*1/*1 (NM_t_)	*1/*1 (NM_t_)	*1/*1 (NM_t_)	*1/*1 (NM_t_)	*1/*5 (NM_t_)	*1/*6 (IM_t_)
	**CYP2C19**	*1/*1 (NM_t_)	*1/*1 (NM_t_)	*1/*2 (IM_t_)	*1/*17 (RM_t_)	*17/*17 (UM_t_)	*1/*17 (RM_t_)	*1/*2 (IM_t_)
	**CYP2D6**	*1/*2Ax2 (UM_t_)	*4/*4 + *68 (PM_t_)	*1/*35 (NM_t_)	*1/*35 (NM_t_)	*1/*2A (NM_t_)	*1/*10 (NM_t_)	*1/*10 + *36 (NM_t_)
**Sex**		Female	Male	Female	Female	Female	Male	Male
**Age (years)**		>60	40-60	20-40	40-60	40-60	>60	<20
**BMI (categories)**		Normal weight	Obesity type I	Underweight	Obesity type II	Overweight	Normal weight	Overweight
**Smoking Habits**		Non-smoker	Smoker	Non-smoker	Non-smoker	Smoker	Non-smoker	Non-smoker
**Sertraline Dose (mg/day)**		200	150	200	100	150	200-300	100
**CSER (ng/mL), Median (IQR)**		87.5 (74–101)	63.1 (-)	30.85 (24–37.7)	23.4 (-)	40 (36.5-45)	45.3 (27.2- 48.2)	57.28 (-)
CDR (ng/mL*mg), Median (IQR)		0.438 (0.37–0.505)	0.421 (-)	0.154 (0.12–0.189)	0.234 (-)	0.267 (0.243–0.3)	0.178 (0.136–0.181)	0.573 (-)
**CDR/kg** **(ng/mL)/(mg/kg), Median (IQR)**		21.5 (17.95-25.05)	41.73 (-)	8.41 (6.52-10.31)	19.89 (-)	17.97 (16.74-20.22)	12.47 (9.53-12.92)	44.16 (-)
**N-SER/SER, Median (IQR)**		1.95 (1.93-1.97)	1.66 (-)	3.92 (3.5-4.33)	2.21 (-)	2.84 (2.29-2.92)	2.52 (2.48-2.89)	1.97 (-)
**CL/F (mL/min), Median (IQR)**		1,626.01 (1,375.14−1,876.88)	1,650.82 (-)	4,735.55 (3,684.06−5,787.04)	2,967.71 (-)	2,604.17 (2,314.82−2,853.88)	3,908.69 (3,832.48−5,106.21)	1,212.37 (-)
**Liver function**		Within range	Within range	Within range	Within range	Within range	Within range	Above range
**Psychiatric diagnoses**		Depressive disorders; Cognitive impairment and dementia	Depressive disorders; Hyperalgesia and opioid-induced memory impairment	Depressive disorders; Eating disorders; Developmental disorders	Depressive disorders; Anxiety disorders	Bipolar disorders; Anxiety disorders; Alcohol, sedative, hypnotic, or anxiolytic related disorders; Accentuation of personality traits	Depressive disorders; Accentuation of personality traits	Psychotic disorder
**Concomitant medication**		Bupropion, mirabegron; other concomitant medications including benzodiazepines, antipsychotics, laxatives, PPI, prokinetics, analgesics, and vitamins.	Bupropion; other concomitant medications including antidepressants, benzodiazepines, antipsychotics, cardiovascular drugs, urological agents, analgesics, and PPI.	Prednisone; other medications include antiepileptics, antidepressants, benzodiazepines, PPI, and respiratory agents.	No concomitant medications with known relevant CYP interactions; other medications include antiepileptics, antidepressants, benzodiazepines, cardiovascular drugs, PPI, and antipsychotics.	No concomitant medications with known relevant CYP interactions; other medications include mood stabilizers, antipsychotics, benzodiazepines, prokinetics, laxatives, and vitamins.	Bupropion; other concomitant medications including antidepressants, benzodiazepines, antipsychotics, cardiovascular drugs, urological agents, lipid-lowering agents, and laxatives.	Olanzapine; other medications include benzodiazepines, antipsychotics, and central nervous system agents.

BMI: body mass index; categories according to the WHO: Underweight: BMI < 18.5, Normal weight: BMI = 18.5–24.9, Overweight: BMI = 25–29.9, Obesity type I: BMI = 30–34.9, Obesity type II: BMI = 35–39.9, Obesity type III: BMI ≥ 40; CDR: concentration/dose ratio; CDR/kg: weight-normalized CDR; CL/F: apparent total clearance; CSER: basal serum concentrations of sertraline; IQR: interquartile range; N-SER/SER: metabolite/parent compound ratio; SD: standard deviation. Genotype-predicted phenotypes (t) reflect expected enzymatic activity based on allele function. IM (intermediate metabolizer); NM (normal metabolizer); PM (poor metabolizer); RM (rapid metabolizer); UM (ultra-rapid metabolizer). Liver function was classified based on AST/ALT relative to institutional reference ranges. To minimize the risk of re-identification, selected demographic and clinical variables are aggregated, and only concomitant medications relevant to CYP-mediated metabolism are reported individually.

Given the naturalistic design, patients frequently presented with multiple psychiatric diagnoses, resulting in a total of 13 different psychiatric diagnoses across the seven individuals. The primary indications for sertraline treatment were depressive and affective disorders (34.1%), accentuation of personality traits without disorder (19.5%), substance use disorders (14.6%), personality or conduct disorders (9.8%), anxiety and acute psychotic disorders (4.9% each), and opioid-induced hyperalgesia or memory impairment (2.4% each). Among depressive disorders, recurrent depressive disorder (ICD-10 code F33) accounted for 50%, major depressive episode without psychotic symptoms (ICD-10 code F32.2) for 28.6%, and bipolar disorder, current episode depressive (ICD-10 code F31.4) for 21.4%. A total of 33 different somatic comorbidities were recorded, most commonly dyslipidemia (12.7%), arterial hypertension (9.9%), benign prostatic hyperplasia (7%), and generalized epilepsy or constipation (4.2%).

Median daily sertraline dose was 200 mg (range 50–300 mg), with a median duration of treatment at stable dose of 9.5 days (IQR 6–17.5). The median CSER was 45.15 ng/mL (IQR 31.85–57.28), CDR 0.239 ng/mL*mg (IQR 0.179–0.395), CDR/kg 17.34 (ng/mL)/(mg/kg) (IQR 11.39–22.63), N-SER/SER ratio 2.38 (1.97–2.91), and CL/F 2,910.8 mL/min (1,763.85–3,870.58). Most observed concentrations fell within the lower–mid range of the AGNP-reported therapeutic window (10–150 ng/mL). As illustrated in Table 3, concomitant medications with potential enzymatic effects included bupropion (potent CYP2D6 inhibitor) and mirabegron (moderate CYP2D6 inhibitor). Based on observed pharmacokinetic patterns and limited literature, bupropion and olanzapine were considered potential contributors to altered CYP2B6-mediated metabolism and prednisone to CYP2C19-mediated metabolism; however, these observations are descriptive and hypothesis-generating.

**Table 3:** Observed genotypes of CYP2B6, CYP2C19, CYP2D6 isoforms, genotype- predicted phenotypes, and descriptively co-medication–adjusted phenotypes [n = 7 patients].

Patient	Genotype			Genotype-predicted phenotypes (_t_)			Co-medication effect	Level of evidence	Descriptively co-medication–adjusted (_p_)		
	CYP2B6	CYP2C19	CYP2D6	CYP2B6	CYP2C19	CYP2D6			CYP2B6	CYP2C19	CYP2D6
A	*6/*6	*1/*1	*1/*2Ax2	PM_t_	NM_t_	UM_t_	Bupropion (strong CYP2D6 inhibitor; possible CYP2B6 interaction) Mirabegron (moderate CYP2D6 inhibitor)	CYP2D6 inhibition: FDA/Flockhart (established) CYP2B6 interaction: literature (limited evidence) CYP2D6 moderate inhibition: FDA/Flockhart (established)	PM_p_	NM_p_	PM_p_
B	*1/*1	*1/*1	*4/*4 + *68	NM_t_	NM_t_	PM_t_	Bupropion (strong CYP2D6 inhibitor; possible CYP2B6 interaction)	CYP2D6 inhibition: FDA/Flockhart (established) CYP2B6 interaction: literature (limited evidence)	IM_p_	NM_p_	PM_p_
C	*1/*1	*1/*2	*1/*35	NM_t_	IM_t_	NM_t_	Prednisone (possible CYP2C19 induction)	Not classified in FDA; literature/ Flockhart (limited evidence)	NM_p_	NM_p_	NM_p_
D	*1/*1	*1/*17	*1/*35	NM_t_	RM_t_	NM_t_	–	–	NM_p_	RM_p_	NM_p_
E	*1/*1	*17/*17	*1/*2A	NM_t_	UM_t_	NM_t_	–	–	NM_p_	UM_p_	NM_p_
F	*1/*5	*1/*17	*1/*10	NM_t_	RM_t_	NM_t_	Bupropion (strong CYP2D6 inhibitor; possible CYP2B6 interaction)	CYP2D6 inhibition: FDA/Flockhart (established) CYP2B6 interaction: literature (limited evidence)	IM_p_	RM_p_	PM_p_
G	*1/*6	*1/*2	*1/*10 + *36	IM_t_	IM_t_	NM_t_	Olanzapine (possible CYP2B6 interaction)	Literature (limited evidence)	PM_p_	IM_p_	NM_p_

Genotype-predicted phenotypes (_t_) reflect expected enzymatic activity based on allele function. Co-medication–adjusted phenotypes (_p_) represent a descriptive clinical framework incorporating potential inhibitory or inductive effects of concomitant drugs and do not constitute direct measurements of enzymatic activity. Perpetrator classification reflects a descriptive approach integrating established interaction resources (FDA and Flockhart tables) and available literature, and should be interpreted according to the indicated level of evidence. Enzymatic phenotype: IM (intermediate metabolizer); NM (normal metabolizer); PM (poor metabolizer); RM (rapid metabolizer); UM (ultra-rapid metabolizer).

### Influence of genotype on sertraline pharmacokinetics

#### Without consideration of phenoconversion.

When pharmacokinetic measurements were grouped according to genotype-predicted phenotypes without accounting for concomitant medications, CYP2B6-related groups showed observable differences across several pharmacokinetic parameters (Table 4). Differences were observed in CSER, CDR, CDR/kg, and CL/F differed across CYP2B6 phenotype categories (descriptive p-values 0.023; 0.018; 0.045; and 0.018 respectively), whereas the N-SER/SER ratio did not show relevant variation (p = 0.139). Median CSER values in CYP2B6 PM_t_ (patient A, n = 2 measurements) were higher than those observed in NM_t_ measurements (patients B-F, n = 12 measurements) (87.5 vs 38.85 ng/mL, descriptive p = 0.023). Median CDR values were higher in PM_t_ (0.438 ng/mL*mg) and IM_t_ (0.573 ng/mL*mg, n = 2 measurements) compared with NM_t_ (0.191 ng/mL*mg; descriptive p-values 0.045 and 0.028). Median CL/F values were lower in PM_t_ (1,626 mL/min) and in IM_t_ (1,212.37 mL/min) compared with NM_t_ (3,642.97 mL/min; descriptive p-values 0.045 and 0.028) and median CDR/kg values were higher in IM_t_ (44.16 (ng/mL)/(mg/kg)) compared with NM_t_ (13.74 (ng/mL)/(mg/kg), descriptive p = 0.028) (Table 4). These comparisons are presented for descriptive purposes and should not be interpreted as inferential, given the small number of individuals and the presence of repeated measurements within individuals.

**Table 4:** Distribution of sertraline pharmacokinetic parameters across CYP2B6 phenotype groupings, without and with descriptive consideration of phenoconversion [n = 16 sertraline measurements].

Pharmacokinetic parameter	Without consideration of phenoconversion				Including descriptive consideration of phenoconversion			
	CYP2B6 genotype (Genotype-predicted phenotypes (_t_))			Descriptive p-value	CYP2B6 genotype (Co-medication–adjusted phenotypes (_p_))			Descriptive p-value
	*6/*6 (PM_t_)	*1/*1 (NM_t_) and *1/*5 (NM_t_)	*1/*6 (IM_t_)		*6/*6 (PM_p_)	*1/*1 (NM_p_) and *1/*5 (NM_p_)	*1/*6 (IM_p_)	
	n = 2	n = 12	n = 2		n = 4	n = 6	n = 6	
**CSER (ng/mL), Median (IQR)**	87.5 (74–101)	38.85 (25.6-46.75)	57.3 (-)	0.023*	65.64 (57.28–87.5)	37.1 (24–40)	46.75 (27.2-53.3)	0.016*
CDR (ng/mL*mg), Median (IQR)	0.438 (0.37-0.505)	0.191 (0.157–0.255)	0.573 (-)	0.018*	0.539 (0.438-0.573)	0.239 (0.189–0.267)	0.179 (0.136-0.193)	0.015*
**CDR/Kg (ng/mL)/(mg/kg), Median (IQR)**	21.5 (17.95-25.05)	13.74 (9.92-18.93)	44.16 (-)	0.045*	34.61 (21.5-44.16)	17.36 (10.31-19.89)	12.7 (9.53-14.58)	0.06
**N-SER/SER, Median (IQR)**	1.95 (1.93–1.97)	2.68 (2.24-3.03)	1.97 (-)	0.139	1.97 (1.95–1.97)	2.88 (2.29-3.5)	2.5 (1.66-2.89)	0.062
**CL/F (mL/min), Median (IQR)**	1,626 (1,375.14–1,876.88)	3,642.97 (2,729.02−4,507.45)	1,212.37 (-)	0.018*	1,293.75 (1,212.37–1,626)	2,910.8 (2,604.17−3,684.06)	3,870.58 (3,601.89−5,106.21)	0.015*

CDR: concentration/dose ratio; CDR/kg: weight-normalized CDR; CL/F: apparent total clearance; CSER: basal serum concentrations of sertraline; IQR: interquartile range; N-SER/SER: metabolite/parent compound ratio. Enzymatic phenotypes are based on genotype-predicted (_t_) or descriptively co-medication-adjusted (_p_) classifications: IM (intermediate metabolizer); NM (normal metabolizer); PM (poor metabolizer). P-values are reported as descriptive measures of between-group differences and should be interpreted cautiously due to the exploratory case-series design and the presence of repeated observations within individuals. Statistical comparisons were performed using the Mann–Whitney U test, *significance at p < 0.05.

For CYP2C19, no relevant differences in pharmacokinetic parameters were observed when analyses were based solely on genotype-predicted phenotypes (Table 5). For CYP2D6, measurements classified as UM_t_ (n = 2) showed higher median CSER values than NM_t_ (n = 13) (87.5 ng/mL vs. 40 ng/mL, descriptive p = 0.027) (Table 6). This apparent pattern suggested a discrepancy between genotype-predicted phenotype and observed exposure, prompting further descriptive evaluation incorporating co-medication data.

**Table 5:** Distribution of sertraline pharmacokinetic parameters across CYP2C19 phenotype groupings, without and with descriptive consideration of phenoconversion [n = 16 sertraline measurements].

Pharmacokinetic parameter	Without consideration of phenoconversion					Including descriptive consideration of phenoconversion				
	CYP2C19 genotype (Genotype-predicted phenotypes (_t_))				Descriptive p-value	CYP2C19 genotype (Co-medication–adjusted phenotypes (_p_))				Descriptive p-value
	*1/*1 (NM_t_)	*1/*2 (IM_t_)	*1/*17 (RM_t_)	*17/*17 (UM_t_)		*1/*1 (NM_p_)	*1/*2 (IM_p_)	*1/*17 (RM_p_)	*17/*17 (UM_p_)	
	n = 3	n = 4	n = 6	n = 3		n = 5	n = 2	n = 6	n = 3	
**CSER (ng/mL), Median (IQR)**	74 (63.1–101)	47.49 (30.85–57.3)	36.3 (23.4–48.2)	40 (36.5–45)	0.058	63.1 (37.7–74)	57.28 (–)	36.3 (23.4–48.2)	40 (36.5–45)	0.215
CDR (ng/mL*mg), Median (IQR)	0.421 (0.37–0.505)	0.381 (0.154–0.573)	0.179 (0.136–0.193)	0.267 (0.243–0.3)	0.067	0.37 (0.189–0.421)	0.573 (–)	0.179 (0.136–0.193)	0.267 (0.243–0.3)	0.034*
**CDR/Kg (ng/mL)/(mg/kg), Median (IQR)**	25.05 (17.95-41.73)	27.24 (8.41–44.16)	12.7 (9.53 −14.58)	17.97 (16.74- 20.22)	0.252	17.95 (10.31-25.05)	44.16 (–)	12.7 (9.53 −14.58)	17.97 (16.74- 20.22)	0.079
**N-SER/SER, Median (IQR)**	1.93 (1.66–1.97)	2.73 (1.97–3.92)	2.5 (2.21 −2.89)	2.84 (2.29–2.92)	0.147	1.97 (1.93–3.5)	1.971 (–)	2.5 (2.2 −2.89)	2.84 (2.29–2.92)	0.682
**CL/F (mL/min), Median (IQR)**	1,650.82 (1,375.14 −1,876.88)	2,448.21 (1,212.37 −4,735.55)	3,870.59 (3,601.9 −5,106.21)	2,604.17 (2,314.82 −2,853.88)	0.067	1,876.88 (1,650.82 −3,684.06)	1,212.37 (-)	3,870.59 (3,601.9 −5,106.21)	2,604.17 (2,314.82 −2,853.88)	0.034*

CDR: concentration/dose ratio; CDR/kg: weight-normalized CDR; CL/F: apparent total clearance; CSER: basal serum concentrations of sertraline; IQR: interquartile range; N-SER/SER: metabolite/parent compound ratio. Enzymatic phenotypes are based on genotype-predicted (_t_) or descriptively co-medication-adjusted (_p_) classifications: IM (intermediate metabolizer); NM (normal metabolizer); RM (rapid metabolizer); UM (ultra-rapid metabolizer). P-values are reported as descriptive measures of between-group differences and should be interpreted cautiously due to the exploratory case-series design and the presence of repeated observations within individuals. Statistical comparisons were performed using the Mann–Whitney U test, *significance at p < 0.05.

**Table 6:** Distribution of sertraline pharmacokinetic parameters across CYP2D6 phenotype groupings, without and with descriptive consideration of phenoconversion [n = 16 sertraline measurements].

Pharmacokinetic parameter	Without consideration of phenoconversion				Including descriptive consideration of phenoconversion		
	CYP2D6 genotype (Genotype-predicted phenotypes (_t_))			Descriptive p-value	CYP2D6 genotype (Co-medication–adjusted phenotypes (_p_))		
	*1/*35; *1/*2A; *1/*10 and *1/*10 + *36 (NM_t_)	*4/*4 + *68 (PM_t_)	*1/*2Ax2 (UM_t_)		*1/*35; *1/*2A; *1/*10 and *1/*10 + *36 (NM_p_)	*4/*4 + *68 (PM_p_)	Descriptive p-value
	n = 13	n = 1	n = 2		n = 8	n = 8	
**CSER (ng/mL), Median (IQR)**	40 (27.2 - 48.2)	63.1 (-)	87.5 (74–101)	0.031*	38.85 (30.25 - 51.14)	50.75 (36.25-68.55)	0.207
CDR (ng/mL*mg), Median (IQR)	0.193 (0.178 −0.267)	0.421 (-)	0.438 (0.37–0.505)	0.192	0.255 (0.211 −0.436)	0.187 (0.157-0.395)	0.344
**CDR/Kg (ng/mL)/(mg/kg), Median (IQR)**	14.58 (10.31-19.89)	47.73 (-)	21.5 (17.95-25.05)	0.323	18.93 (13.53-32.19)	13.75 (11-21.5)	0.344
**N-SER/SER, Median (IQR)**	2.52 (2.21–2.92)	1.66 (-)	1.95 (1.93–1.97)	0.082	2.56 (2.09–3.21)	2.22 (1.8-2.7)	0.172
**CL/F (mL/min), Median (IQR)**	3,601.89 (2,604.17–3,908.69)	1,650.82 (-)	1,626 (1,375.14–1,876.88)	0.192	2,729.02 (1,763.59–3,325.88)	3,717.18 (1,763.85–4,507.45)	0.344

CDR: concentration/dose ratio; CDR/kg: weight-normalized CDR; CL/F: apparent total clearance; CSER: basal serum concentrations of sertraline; IQR: interquartile range; N-SER/SER: metabolite/parent compound ratio. Enzymatic phenotypes are based on genotype-predicted (_t_) or descriptively co-medication-adjusted (_p_) classifications: NM (normal metabolizer); PM (poor metabolizer); UM (ultra-rapid metabolizer). P-values are reported as descriptive measures of between-group differences and should be interpreted cautiously due to the exploratory case-series design and the presence of repeated observations within individuals. Statistical comparisons were performed using the Mann–Whitney U test, *significance at p < 0.05.

### Including phenoconversion as a descriptive framework

When phenotype groupings were descriptively adjusted to account for concomitant medications, patterns associated with CYP2B6 remained observable across CSER, CDR, and CL/F, while differences in CDR/kg were attenuated (before adjustment descriptive p-value 0.045 and after adjustment p-value 0.06) (Table 4). Measurements classified as CYP2B6 PM_p_ (n = 4) showed higher median CSER and CDR values (65.64 ng/mL and 0.539 ng/mL*mg respectively) and lower CL/F values (1,293.75 mL/min) compared with NM_p_ (n = 6; 37.1 ng/mL; 0.239 ng/mL*mg and 2,910.8 mL/min, descriptive p-values: 0.01) and IM_p_ (n = 6; 46.75 ng/mL; 0.179 ng/mL*mg and 3,870.58 mL/min, descriptive p-values: 0.033; 0.019 and 0.019) (Table 4). These comparisons are presented as descriptive indicators of exposure patterns rather than inferential statistical findings.

For CYP2C19, incorporation of phenoconversion revealed previously unobserved patterns in CDR and CL/F (descriptive p-value 0.034) (Table 5). Measurements classified as IM_p_ (n = 2) showed higher CDR and lower CL/F values (0.573 ng/mL*mg and 1,212.37 ml/min) compared with RM_p_, (n = 6; 0.179 ng/mL*mg and 3,870.59 mL/min, descriptive p-values 0.044) while UM_p_ measurements (n = 3) also exhibited lower CL/F values (2,604.17 ml/min) than RM_p_. The UM_p_ CDR values were higher compared with RM_p_ (0.267 vs 0.179 mg/mL*mg, descriptive p-value 0.02). These findings were unexpected and varied across individuals, underscoring the complexity of interpreting CYP2C19-related variability in the presence of multiple co-medications and potential absorption-related factors. After consideration of phenoconversion, no consistent patterns were observed for CYP2D6 across pharmacokinetic parameters (Table 6), suggesting limited contribution of this isoenzyme to sertraline exposure within this case series.

## Discussion

In psychiatric practice, polypharmacy is frequent due to patient complexity and comorbidities. While pharmacogenetic testing of drug-metabolizing enzymes can inform initial dosing decisions, concomitant medications may also contribute to variability in enzymatic activity, underscoring the need for an integrated and contextualized interpretation. The resulting functional metabolic capacity—often described as a phenoconversion effect—may therefore differ from the genotype-predicted phenotype when inhibitory or inducing co-medications are present [11,13,39,40]. Smoking status was also recorded as a potential modifier of enzymatic activity; however, given the limited exposure in this cohort and the minor role of CYP1A2 in sertraline metabolism, no relevant impact on pharmacokinetic variability was observed. The classification of some perpetrator drugs was based on limited or indirect evidence, and should therefore be interpreted cautiously, particularly where not supported by standard interaction tables. In contrast to prior studies [25,28,33–35,41], the present case-series describes pharmacokinetic patterns that are broadly consistent with variability across individual CYP isoforms in sertraline disposition, integrating genotype information and co-medication context using standard pharmacokinetic parameters.

When each CYP450 isoform was examined separately, CYP2B6 phenotype groupings were associated with differences in CSER, CDR, and CL/F, with lower sertraline exposure and higher apparent clearance observed in NM_p_ compared with PM_p_, and similar trends when compared with IM_p_. These observations are consistent with previous reports describing lower apparent sertraline clearance and higher plasma concentrations in CYP2B6 IM and PM phenotypes compared with NMs [23,24,28,34]. This pattern may partly reflect the distributed and stereoselective metabolism of sertraline across multiple CYP enzymes, which can attenuate the magnitude of single-enzyme effects *in vivo* and contribute to the more modest or inconsistent associations observed for other pathways.

It is important to consider concomitant medications when interpreting genetic information, as they may influence the functional translation of a given genotype. Bupropion, a known potent inhibitor of CYP2D6, has also been described as a possible competitive inhibitor of CYP2B6, although clinical evidence remains limited [12]. This mechanism may help contextualize the phenoconversion patterns observed in patients A, B, and F, although alternative explanations cannot be excluded, potentially affecting both CYP2D6 and CYP2B6. Patients A and B, receiving 150–450 mg/day of bupropion, exhibited CDR values notably higher than the population median (0.438 and 0.421, respectively, vs. 0.239 ng/mL*mg). Although evidence regarding olanzapine as a CYP2B6 inhibitor remains inconclusive, it is notable that olanzapine undergoes extensive first-pass metabolism, with approximately 40% of the administered dose metabolized before reaching systemic circulation [42]. This may lead to under-recognition of the enzymes involved in its biotransformation, as early pharmacokinetic studies did not evaluate CYP2B6 involvement [43,44]. Available population pharmacokinetic data suggest that sertraline may increase olanzapine clearance, while no consistent reciprocal effect of olanzapine on sertraline pharmacokinetics has been demonstrated [45,46]. In this context, the elevated exposure observed in our dataset should be interpreted descriptively, without assuming a directional interaction. Patient G illustrates this scenario: co-treatment with 30 mg/day olanzapine coincided with a shift from genotype-predicted IM_t_ for CYP2B6 and CYP2C19, and NM_t_ for CYP2D6, to descriptively adjusted PM_p_ for CYP2B6, IM_p_ for CYP2C19 and NM_p_ for CYP2D6 phenotypes, accompanied by markedly elevated CDR values (0.573 vs 0.239 ng/mL*mg).

In this dataset, failure to consider phenoconversion would potentially obscure the contribution of CYP2C19 to sertraline pharmacokinetics, despite prior evidence supporting its role [25,28,33–35]. Phenoconversion was descriptively illustrated by patient C, receiving prednisone, for which a potential inductive effect on CYP2C19 has been suggested in clinical observational studies, although not consistently supported by standard interaction classifications [47], who showed a CDR substantially lower than the population median (0.154 vs. 0.239 ng/mL*mg). After descriptive adjustment, IM_p_ exhibited lower clearance than RM_p_ for CYP2C19 (CDR and CL/F), and UM_p_ unexpectedly showed lower clearance than RM_p_. Previous studies suggest that increased-function alleles such as CYP2C19*17 may be less predictive of pharmacokinetic variability than reduced-function alleles [33,35]. This may partially explain the patterns observed between our UM_p_ and RM_p_ patients, where differences in sertraline exposure were not exclusively associated with the phenoconversion-adjusted metabolic phenotype, but also with co-medications capable of variably modifying drug absorption, such as fibre-containing laxatives [34]. This complexity is illustrated by patient F, who displayed an RM_p_ phenotype for CYP2C19, IM_p_ for CYP2B6 and PM_p_ for CYP2D6. Despite the presence of bupropion, whose effect on CYP2B6 remains uncertain, concomitant fibre administration likely reduced sertraline absorption. This patient received 200–300 mg/day of sertraline and exhibited a CL/F value of 3,908 mL/min, markedly higher than the median observed among CYP2C19 UM_p_ patients (2,604 mL/min). This elevated CL/F value is unlikely to solely reflect increased hepatic metabolism and may instead be influenced by reduced drug absorption. After accounting for phenoconversion, CYP2D6 no longer showed an association with sertraline pharmacokinetics, reinforcing prior evidence suggesting limited clinical relevance of this isoform in sertraline metabolism [25,41]. This observation highlights the importance of integrating phenoconversion into pharmacogenetic interpretation, as failure to do so may lead to potentially misleading associations driven by co-medication effects rather than intrinsic metabolic capacity [11,13,34,39–41]. Across all analyses, the N-SER/SER ratio remained unaffected by genotype or phenotype, supporting the notion that CYP3A4 is the principal enzyme responsible for N-SER formation [25].

Although current pharmacogenetic guidelines primarily emphasize CYP2C19 in the context of sertraline therapy [28,34,35], our findings suggest that CYP2B6 may represent a potentially informative component of pharmacogenetic evaluation. After accounting for phenoconversion, CYP2B6 appeared to exert a stronger influence on CSER, CDR, and CL/F than CYP2C19, which primarily affected CDR and CL/F. From a clinical perspective, intermediate and poor metabolizer phenotypes may exert greater influence on sertraline exposure than rapid or ultrarapid phenotypes. Given that increased-function CYP2C19*17 alleles are more prevalent in European populations (approximately 22%) than reduced-function alleles (*2–*4, approximately 18%) [29], CYP2B6 genotyping may offer complementary and potentially greater clinical utility in selected patients. These population differences in allele distribution may contribute to variability in sertraline exposure across clinical settings. While the primary focus of this study is pharmacokinetic variability, such exposure differences may also have clinical relevance in relation to the known safety profile of SSRIs.

In this context, this study was not designed to systematically assess safety outcomes, the observed variability in sertraline exposure should be interpreted in the context of known adverse effects associated with SSRIs, including bleeding risk (particularly with antiplatelet agents), hyponatremia in susceptible populations, and exposure-related QT interval prolongation at higher doses. Systematic safety data (e.g., laboratory values or ECG measurements) were not consistently available in this retrospective cohort, limiting any formal exposure–safety assessment. These aspects should therefore be considered exploratory and highlight the need for future studies integrating pharmacokinetic and safety endpoints in real-world settings.

Several limitations warrant consideration. The small number of pharmacokinetic measurements necessitated the use of non-parametric statistical methods and limited statistical power, a constraint shared by other real-world pharmacokinetic investigations [48]. The retrospective and observational nature of the study precludes causal inference, and repeated measurements within individuals were retained to reflect real-world clinical trajectories rather than to support population-level inference. In addition, variability in the duration of treatment at stable dose prior to sampling, as well as the use of total (rather than unbound) concentrations, and the lack of assessment of transporter-mediated variability (e.g., ABCB1/P-glycoprotein), may have contributed to variability in pharmacokinetic parameters. Furthermore, these findings may not be generalizable to special populations with altered pharmacokinetics, such as pregnancy, where increased variability has been described, and therapeutic drug monitoring may be particularly relevant in selected cases. Accordingly, all findings should be interpreted as exploratory and hypothesis-generating.

Despite these limitations, this study provides supportive evidence for the integration of CYP2B6 genotyping and phenoconversion assessment into pharmacogenetic evaluation of antidepressant therapy. The data suggest that CYP2B6 is likely to be an important CYP450 isoform influencing sertraline exposure, with CYP2C19 exerting a secondary but clinically relevant role once phenoconversion is considered, while CYP2D6 appears to have minimal impact. Importantly, these relationships become apparent when the potential modifying effects of concomitant medications are taken into account. Failure to incorporate phenoconversion may therefore lead to underestimation of CYP2C19’s contribution and overestimation of CYP2D6’s relevance.

## Conclusions

In this exploratory case series, CYP2B6 appeared to be the CYP450 isoform most consistently associated with variability in sertraline pharmacokinetics, with CYP2C19 showing a secondary but potentially relevant contribution once phenoconversion is considered, while CYP2D6 showed minimal influence. These findings support the integration of the patient’s complete medication profile in pharmacogenetic interpretation to reduce the risk of misclassification of metabolic status. The combined application of pharmacogenetic testing and therapeutic drug monitoring may represent a complementary and clinically valuable approach to individualize dosing, improve safety, and support optimization of antidepressant treatment strategies.
